# Transmitted HIV-1 variants in HIV infected mother-child pairs carrying different subtypes

**DOI:** 10.1186/1742-4690-9-S2-P162

**Published:** 2012-09-13

**Authors:** R Caridha, K Gieng, U Fried, S Lindgren, P Clevestig, A Ehrnst

**Affiliations:** 1 Karolinska Institutet, Stockholm, Sweden; 2 Karolinska Institutet, Unit of Obstetrics and Gynecology, Huddinge, Stockholm, Sweden

## Background

Mother-to-child transmission is the predominant route of HIV-1 infection in children. Prevention strategies are available in industrialized countries, but are limited in developing countries. Defining pertinent characteristics of transmitted HIV-1 from mother-child pairs, where the actually transmitted virus has been traced, is important in creating better preventive measures against transmission of HIV-1.

## Methods

Two mother-child pairs of subtype A, three of subtype C, and three of CRF01_AE, were included. The V3-loop and flanking regions of the HIV-1 env gene were amplified from PBMC and plasma lysates. Virus isolates from several time points of pregnancy and in the child were sequenced, as well as 50 to 100 single HIV genomes per case.

## Results

A total of 543 single genome sequences were obtained. The sequences were analysed phylogenetically, demonstrating at least one maternal sequence, identical to the child's first detected viruses or clones. In all cases an infant isolate sequence co-localized with maternal sequences, most probably related to transmission. The localization of other sequences from clones and/or virus isolates in the phylogenetic tree and their detailed amino acid composition provided a clear indication of the V3 loop amino acids of the respective transmitted virus. The fact that these sequences included an isolate will allow a greater genetic analysis of the complete envelope of transmitted HIV-1.

## Conclusion

Since the sequence of the gp120 V3 loop is of paramount importance for virus entry, we can compare the amino acid properties across subtypes and test these viruses in neutralization assays. This knowledge can be exploited in prevention strategies and vaccine design.

